# The Release Characteristic and Removal of Heavy Metal and HCl During Co-Combustion of MSW and Aged Refuse: A Preliminary Study Based on Thermodynamic Equilibrium Analysis

**DOI:** 10.3390/molecules30244771

**Published:** 2025-12-14

**Authors:** Limei Chen, Yaojie Wang, Yanfen Liao, Xiaoqian Ma

**Affiliations:** 1 School of Automation, Guangdong Polytechnic Normal University, Guangzhou 510665, China; 2 Building Equipment Information Integration and Control Key Laboratory, Guangzhou 510665, China; 3 School of Electric Power, South China University of Technology, Guangzhou 510640, China

**Keywords:** heavy metals, HCl, thermodynamic equilibrium analysis, calcium-based additives, aged refuse

## Abstract

Co-combustion in a refuse incinerator is a primary method for treating aged refuse (AR). Given the high contents of heavy metals and chlorine in AR, it is crucial to investigate their release and fate during co-combustion to achieve environmentally sound treatment. This study investigated the release and volatilization of heavy metals (Cd, Cr, Zn, Ni, Cu, Pb) and HCl during the co-combustion of AR and municipal solid waste (MSW) through chemical thermodynamic equilibrium analysis. The effects of several parameters on the volatilization of heavy metals and HCl were analyzed, including incineration temperature, the N_2_/O_2_ ratio, the degree of refuse classification, the blending ratio of AR, and the effects of conventional calcium-based additives. The results showed that high temperature promoted the volatilization of Cd, Pb, Cu, Ni, and HCl. A lower N_2_/O_2_ ratio suppressed Zn and HCl volatilization. A higher degree of MSW classification (with lower proportions of kitchen and wood waste) and an increased AR blending ratio enhanced Zn fixation. CaO at high temperature only suppressed HCl volatilization, with a minor effect on heavy metals. Two modified calcium-based additives (CaBSiO_4_OH and CaB_5_SiO_9_(OH)_5_) with strong high-temperature Cu removal capabilities were explored, and their risk index was analyzed.

## 1. Introduction

The continuous economic and social development has led to a steady increase in MSW generation. Landfilling remains the primary method for MSW disposal in many developing countries [1]. In recent years, however, the economic viability of landfilling has diminished due to the aging of landfill sites, population growth, and land scarcity [2]. Therefore, identifying effective approaches for managing AR in landfills is crucial. AR, particularly material that has been landfilled for over a decade, has undergone the degradation of its easily decomposable components, resulting in almost no leachate, landfill gas, or odor [3]. Excavating AR that is suitable for mining can free up landfill space and extend the site’s operational lifespan. China’s ongoing promotion of refuse classification is reducing the amount of MSW destined for incineration, thereby creating surplus capacity that can be used for the co-combustion of AR [4]. Utilizing the excess incineration capacity of thermal power plants provides an effective means for disposing of the combustible components of AR.

Plastics, which contain a high chlorine content, account for more than 70% of the combustible fraction in AR [5]. During combustion, the corrosive gas HCl is generated, which damages heating surfaces and causes environmental pollution. Furthermore, AR contains elevated levels of heavy metals [6], which can volatilize into the flue gas during combustion, posing an environmental threat. Moreover, the presence of chlorine has been shown to promote the volatilization of heavy metals [7], exacerbating environmental pollution. Consequently, the high chlorine content in AR significantly contributes to the migration of heavy metals. Therefore, it is necessary to study the release characteristics of gaseous heavy metals and HCl and to effectively remove HCl and heavy metals during the co-combustion of AR in thermal power plants.

Several studies have investigated the co-combustion of AR with other fuels, as well as the removal of heavy metals and chlorine. Wang [8] investigated the retention capacities of natural and modified diatomite for heavy metals during the co-combustion of AR and MSW through a tube furnace experiment. Their results showed that natural diatomite had the highest retention performance at 850 °C, while Na+-modified diatomite exhibited better removal efficiency for certain heavy metals at 700 °C. However, this study is limited to the temperature range of 700–900 °C and does not investigate the retention behavior at temperatures below 700 °C or above 900 °C. Additionally, the retention capacity of the modified diatomite at high temperatures was limited and was effective only at 700 °C. This indicates a need for more effective additives at temperatures exceeding 700 °C. Li [9] investigated the effects of the combustion ratio and the combustion atmosphere on heavy metal volatilization during the co-combustion of MSW with AR. They found that increasing the oxygen content in the combustion atmosphere reduced the volatilization of Cr and Mn. However, the blending ratio of AR in their study was relatively low. Given that many incineration plants in China operate below capacity, this low blending ratio does not reflect the conditions or heavy metal volatilization associated with higher AR blending ratios. Krol [10] examined the volatilization of heavy metals (Cu, Ni, Pb) and Cl during the combustion of plastic, pharmaceutical, and pesticide wastes. They also tested the effectiveness of additives such as V_2_O_5_, Na_2_B_4_O_7_, and their mixtures with CaO in removing heavy metals and chlorine during combustion. The results indicated that at 1273K, a mixture of V_2_O_5_ and CaO reduced Pb emissions during combustion from plastic waste by approximately 65% and from pesticide waste by about 40%. However, the study was limited to temperatures of 1273 K and 1373 K and covered a limited range of heavy metal types. In summary, there is a clear need for further research under a wider range of combustion conditions, including broader temperature ranges and higher blending ratios of AR. Furthermore, conventional fixatives exhibit weak fixation capacity across critical temperature ranges and low effectiveness in removing certain heavy metals. Consequently, developing novel fixatives with superior performance is essential to achieve efficient co-removal of heavy metals and HCl.

Therefore, this paper investigates the migration behavior of heavy metals (Cd, Cr, Zn, Ni, Cu, Pb) and HCl during the co-combustion of AR and MSW using thermodynamic equilibrium analysis. The study focuses on analyzing the effects of key parameters, including incineration temperature, oxygen-to-nitrogen ratio, degree of refuse classification, blending ratio of AR, and the use of conventional calcium-based additives, on the volatilization rates of heavy metals and chlorine. In summary, there is a clear need for further research under a wider range of combustion conditions, including broader temperature ranges and higher blending ratios of AR. Furthermore, conventional fixatives exhibit weak fixation capacity across critical temperature ranges and low effectiveness in removing certain heavy metals. Consequently, developing novel fixatives with superior performance is essential to achieve efficient co-removal of heavy metals and HCl. Thermodynamic simulation can provide critical insights for developing modified additives and significantly reduce associated economic costs.

## 2. Materials and Methods

The Equilib module in FactSage 7.3 was employed for the thermodynamic equilibrium calculations. This module is based on the principle of Gibbs free energy minimization. The initial reaction components, temperature, and pressure were specified as input parameters for the calculations. The system was treated as a closed ideal system, where the total Gibbs free energy is minimized at thermodynamic equilibrium for multiphase and multicomponent systems. This calculation focuses solely on the final result at equilibrium, without accounting for the residence time required to reach this state. The following FactSage 7.3 databases were selected: FactPS, FToxid and FTpulp. The composition of the refuse and the atmosphere was converted into elemental input forms [11]. The calculation results were screened to remove negligible products. Only the products constituting more than 1% of the original input mass for each respective element were retained.

### 2.1. Materials

MSW was classified into the following categories: kitchen waste, wood, bamboo, paper, rubber, plastic, and textile. The elemental contents of C, H, O, N, S, Cl, heavy metals, and alkali metals from the bottom ash in MSW were obtained from previous studies [12,13,14,15,16]. Based on the refuse classification level, MSW was classified into three categories: high degree classification, medium degree classification, and low degree classification, with their specific compositions detailed in Table 1 [17]. As the classification level of MSW increased, the proportions of kitchen waste and wood decreased, while those of other waste types increased. The elemental composition (C, H, O, N, S, Cl, heavy metals, and alkali metals from the bottom ash) for each refuse classification was calculated based on the composition of its individual components. This methodology was also applied to determine the heavy metal composition of AR. The input parameters for AR and MSW at different classification degrees, derived from literature data and presented in Table 2, include the contents (wt%) of C, H, O, N, S, Cl, K, Na, Mg, Ca, Al, Fe, and H_2_O, and the contents (mg/kg) of selected heavy metals including Pb, Cd, Cr, Cu, Zn, and Ni.

### 2.2. Research Methods

To investigate the effects of incineration temperature, the proportion of nitrogen to oxygen (N_2_/O_2_), the degree of MSW classification, the blending ratio of AR, and the additives on the migration of heavy metals and HCl, a series of thermodynamic equilibrium calculations was performed under the following conditions. (1) Theoretical air volume (defined as the stoichiometric air required for complete combustion of combustible elements like C, H, N, and S, calculated based on elemental composition, i.e., excess air ratio λ = 1) with an N_2_/O_2_ ratio of 8:2, a 30% blending ratio of AR with MSW (medium degree classification), and a temperature range of 300–1300 °C. (2) A temperature of 800 °C and a 30% blending ratio of AR with MSW (medium degree classification), with N_2_/O_2_ ratios of 7:3, 8:2, and 9:1 (λ = 1). (3) Theoretical air volume (λ = 1) with an N_2_/O_2_ ratio of 8:2, a temperature of 800 °C, a 30% blending ratio of AR, and varying degrees of MSW classification (high, medium, low). (4) Theoretical air volume (λ = 1) with an N_2_/O_2_ ratio of 8:2, a temperature of 800 °C, and AR blending ratios of 0%, 30%, 50%, 70%, 90%, and 100%. (5) The baseline conditions (scenario 1) with the addition of 5% CaO and a modified calcium-based additive.

### 2.3. Ecological Risk Analysis Method

The single potential ecological risk factor for heavy metals volatilized into the gas phase was adapted from Lars Hakanson’s method for water pollution [19], following its validation for assessing flue gas heavy metals in our prior work [20], Huang [21], and You [22] and is defined as follows:

Er = T_r_C_i_/C_r_ (1)

where Er is a single potential ecological risk factor for gaseous heavy metals; T_r_ is a toxic reaction factor of heavy metals; C_i_ is the measured concentration of heavy metals; C_r_ is the concentration of heavy metals in the atmosphere, μg/m^3^.

The T_r_ values assigned for Pb, Cd, Cr, Cu, Zn and Ni were 5, 30, 2, 5, 1 and 5, respectively. C_r_ was the average heavy metal concentration in the atmosphere of cities in China. The corresponding Cr for Pb, Cd, Cr, Cu, Zn and Ni were 0.3373, 0.0071, 0.0636, 0.0817, 0.6762 and 0.0436, respectively [23]. C_i_ was the quotient of the heavy metal content in the gas phase and the volume of gas entering the atmosphere.

Heavy metal pollution was assessed using the risk index (RI), which was calculated as the sum of the individual Er factors for all heavy metals studied:

RI = ∑Er(2)

## 3. Results and Analysis

### 3.1. Influence of Temperature on the Migration Behavior of Heavy Metals and HCl

**Figure 1:**
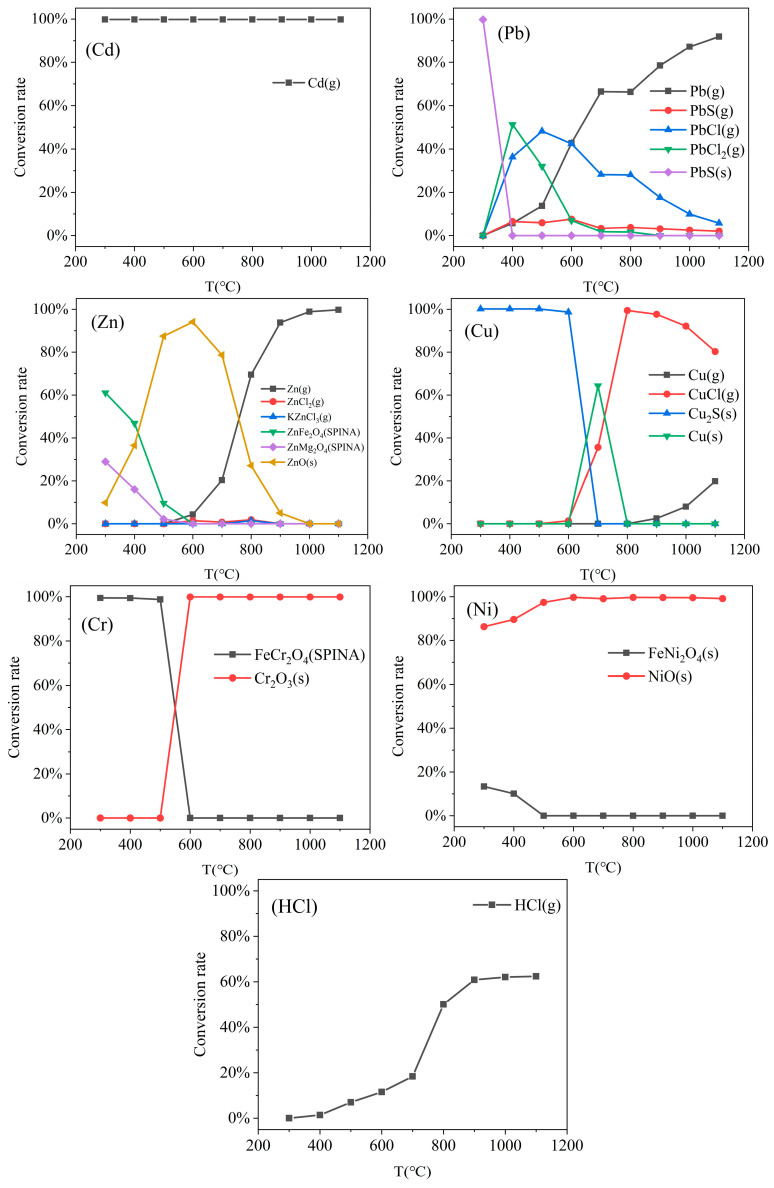
Effect of temperature on the equilibrium speciation fractions of HCl and the products of Cd, Pb, Zn, Cu, Cr, and Ni.

The effect of temperature on the migration behavior of heavy metals is illustrated in Figure 1. Cd exhibits significant volatility across the entire simulated temperature range. Starting from 300 °C, the conversion rate of Cd exceeds 99.79%, indicating near-complete conversion with little variation over the temperature range. This phenomenon indicates that Cd is largely converted into gaseous Cd(g) at lower temperatures, likely owing to such factors as its low boiling point, low Gibbs free energy, and high saturated vapor pressure [24]. Consequently, Cd tends to exist primarily as elemental vapor rather than as oxides or chlorides.

The migration behavior of Pb is characterized by distinct stages in different temperature intervals. At 300 °C, Pb is primarily immobilized as PbS(s) in the bottom ash. Within the 400–800 °C range, however, the formation of PbCl(g) and PbCl_2_(g) increases significantly, accounting for 36.3% and 51.3%, respectively, at 400 °C. This indicates that chlorine significantly promotes Pb volatilization under medium-temperature conditions. Chen [25] conducted thermogravimetric analysis between 783 and 953 K, revealing that the activation energy for PbS chlorination is only 15.3 kJ/mol. This suggests that the reaction is controlled by Cl_2_ diffusion. The volatilization rate of the formed PbCl_2_(g) also increases markedly with temperature. This trend further validates the promoting role of Cl in Pb volatilization within the medium-temperature range. Above 900 °C, the conversion to PbCl(g) and PbCl_2_(g) virtually ceases, whereas that of PbS(g) also decreases. This indicates that Pb chlorides and sulfides decompose at high temperatures, transforming into the more stable gaseous elemental Pb(g).

Within the 300–400 °C range, Zn primarily reacts with metals in the bottom ash to form solid solution phases such as ZnFe_2_O_4_(SPINA) and ZnMg_2_O_4_(SPINA), with a small amount of ZnO(s) detected. As the temperature rises to 500–800 °C, molten zinc compounds in the bottom ash largely convert to ZnO(s), accompanied by trace formation of ZnCl_2_(g) and KZnCl_3_(g). Jenny [26] characterized the speciation of Zn in fly ash using X-ray absorption spectroscopy (XAS), finding that it primarily exists as oxides, which is in close agreement with the simulation results of this study. When the temperature exceeds 900 °C, both zinc chlorides and oxides undergo extensive conversion to gaseous Zn(g).

At 600 °C, Cu primarily exists as Cu_2_S in the bottom ash. When the temperature rises to 700 °C, Cu temporarily converts to Cu(s) and begins to form CuCl(g). With further heating, CuCl(g) becomes the predominant volatile species, accompanied by the volatilization of a small amount of Cu(g). This indicates that Cu readily reacts with Cl at elevated temperatures to form volatile chlorides. This result aligns with the findings of Sorum [27] on copper behavior under grate furnace conditions, which demonstrated Cu’s strong tendency toward chlorination in oxidizing environments.

Within the simulated temperature range, Cr exists exclusively in solid form. Below 500 °C, Cr primarily occurs as FeCr_2_O_4_(s) in the bottom ash. However, when the temperature exceeds 600 °C, it is completely converted to Cr_2_O_3_(s). Therefore, given its low volatility, Cr is classified as a low-volatility heavy metal. Under high-temperature conditions, it stabilizes as Cr^3+^ within the oxide lattice, which effectively immobilizes Cr and leads to its predominant accumulation in the bottom ash. Yang [28], using multiple characterization techniques, observed that Cr combines with various metals in bottom ash at low temperatures, whereas at high temperatures, it predominantly exists as hexagonal plate-like Cr_2_O_3_ crystals. Ni similarly persists in solid phase throughout the temperature range, primarily as NiO(s). Below 500 °C, a portion of Ni forms FeNi_2_O_4_(s) with Fe in the bottom ash. By 600 °C, however, it is almost entirely converted to NiO(s). Zhang [29], based on analysis of waste incinerator bottom ash, found that Ni is not readily volatilized and migrates mainly via particle entrainment, with more than 80% retained in the bottom ash.

The conversion rate of HCl exhibits an overall increasing trend with temperature. At temperatures below 600 °C, HCl production remains low. This is primarily because chlorine release from organic compounds is incomplete, and chlorine may be retained in solid chloride forms. When temperatures exceed 700 °C, the HCl volatilization rate rises significantly. This increase is likely attributed to the thermal decomposition of organic chlorides and the reaction of inorganic chlorides (e.g., NaCl, KCl) with acidic gases (e.g., SO_2_) under high-temperature conditions, which collectively enhance gaseous HCl formation [30,31]. High temperatures not only accelerate the release of Cl but also enhance the stability of HCl in the gas phase, leading to a significant increase in its conversion rate. Above 900 °C, the volatilization rate of HCl tends to stabilize.

In practical municipal solid waste incineration systems, maintaining excess air (λ > 1) is generally necessary to ensure complete combustion and effectively control pollutant formation. When λ = 1, the oxygen supply is primarily consumed in the combustion of carbon and hydrogen, which may lead to the formation of reduction products in localized zones, such as PbS(s) and Cu_2_S(s). Through thermodynamic calculations and experimental studies, Liu [32] and Enestam [33] have confirmed that heavy metal sulfides can form during combustion and contribute to the immobilization of heavy metals. In engineering practice, however, the presence of sulfides is not commonly observed. To further compare the effects of excess air on heavy metal migration under realistic engineering conditions, this study examines the volatilization characteristics of HCl and heavy metals at λ = 1.2 and compares them with those at λ = 1. The results, presented in Table 3, indicate that excess air enhances HCl volatilization at lower temperatures while suppressing it at higher temperatures. It also enhances Cu and Pb volatilization at low temperatures but suppresses Cd volatilization in the 300–400 °C range. Moreover, excess air significantly suppresses Zn volatilization at high temperatures, whereas Ni volatilization is largely unaffected and is retained mainly in the bottom ash. These changes result primarily from an enhanced oxidizing atmosphere: excess oxygen promotes the conversion of Zn, Cu, and Pb into more stable oxides, enhancing Zn fixation at high temperatures but reducing Cu fixation at lower temperatures. Supplementary Figure S1 shows the equilibrium distribution of various heavy metal species as a function of temperature.

### 3.2. Influence of Nitrogen to Oxygen Ratio on Migration Characteristics of Heavy Metals and HCl

**Figure 2:**
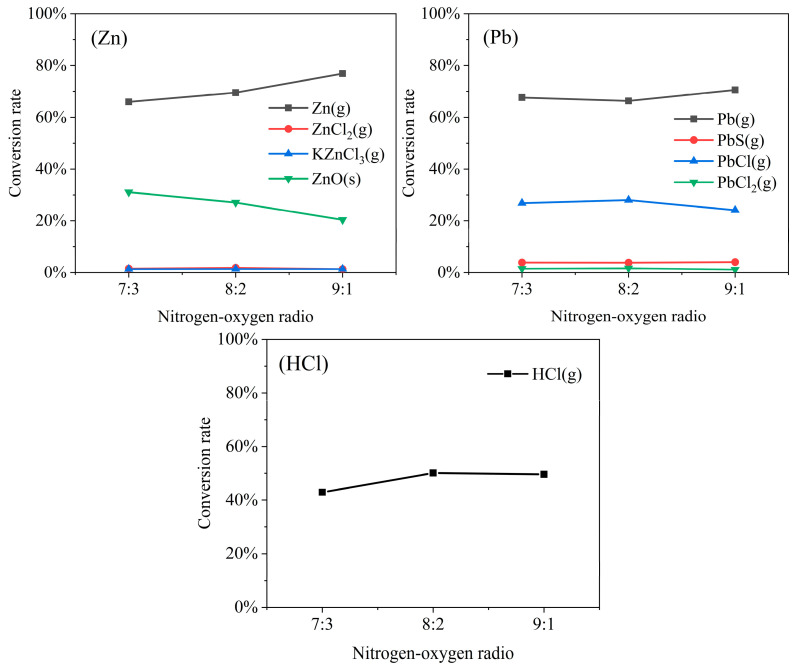
Effect of N_2_/O_2_ ratio on the equilibrium speciation fractions of HCl and the products of Zn and Pb.

This section analyzes the simulation results from condition (2) to investigate how the N_2_/O_2_ ratio affects the speciation of heavy metals. As shown in Figure 2, the N_2_/O_2_ ratio significantly affects the speciation and volatility of Zn, Pb, and HCl. Supplementary Figure S2 shows that virtually no changes were observed in the volatilization of Cd, Ni, and Cu or in the yields of their volatile products. This observation can be attributed to the inherently low volatility of these metals or the limited variety of volatile products formed at this temperature.

At 800 °C, the N_2_/O_2_ ratio significantly affected the transformation of Zn species. As the oxygen proportion decreased (higher N_2_/O_2_ ratio), the conversion rate of Zn(g) gradually increased from 65.9% to 76.9%, while that of ZnO(s) correspondingly decreased from 31.1% to 20.4%. These results indicate that higher N_2_/O_2_ ratios promote the volatilization of Zn as metallic vapor rather than its retention as a stable oxide. Although chlorine is present in the system, the amounts of ZnCl_2_(g) and KZnCl_3_(g) formed remained low, indicating that chlorination is not the primary pathway for Zn volatilization under these conditions. This phenomenon is consistent with the observations reported by Li [9] in their study on AR co-combustion under different N_2_/O_2_ ratios, where a higher N_2_/O_2_ ratio was found to reduce the degree of Zn fixation in the bottom ash. At 800 °C, Pb existed primarily in volatile forms. Under higher N_2_/O_2_ ratios, Pb volatilization as Pb(g) was promoted, while the formation of PbCl(g) was suppressed. The concentrations of PbCl_2_(g) and solid Pb(s) showed no significant change. Furthermore, at an N_2_/O_2_ ratio of 7:3, the volatilization rate of HCl decreased by 7.18%. Collectively, these results demonstrate that maintaining a higher oxygen concentration (i.e., a lower N_2_/O_2_ ratio) is an effective strategy to mitigate the release of volatile Zn and HCl.

### 3.3. Influence of MSW Classification Degree on Migration Characteristics of Heavy Metals and HCl

**Figure 3:**
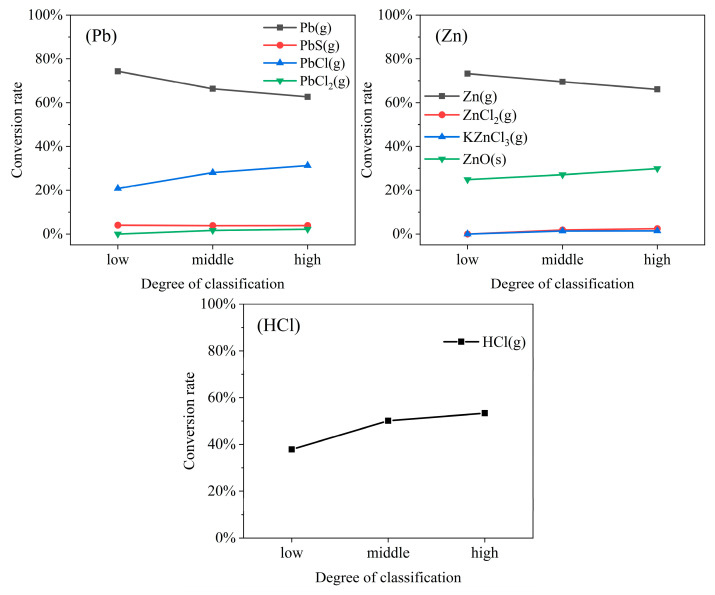
Effect of refuse classification degree on the equilibrium speciation fractions of HCl and the products of Zn and Pb.

MSW classification has become a cornerstone of modern refuse management and recycling systems. To investigate the influence of the MSW classification degree on heavy metal speciation, the simulation results from condition (3) were analyzed. As shown in Figure 3, the degree of MSW classification significantly affects the speciation of Pb, Zn, and HCl. No significant effect on the volatilization of Cr, Ni, Cd, and Cu was observed, as shown in Supplementary Figure S3.

As the degree of refuse classification increases, the proportion of food waste and wood and bamboo in the feedstock decreases, whereas the proportion of rubber, plastics, and other chlorinated materials increases. This change in feedstock composition significantly affects the migration behavior of heavy metals and HCl release during combustion. Notably, the volatilization behavior of Zn and Pb was significantly affected, and as the degree of MSW classification increased, it led to decreased conversion rates of the elemental species Zn(g) and Pb(g) and increased conversion rates of their chloride forms, ZnCl_2_(g), KZnCl_3_(g), PbCl(g), and PbCl_2_(g). This transformation in speciation and the steady increase in HCl release exhibited a marked synergistic effect. This suggests that higher chlorine content in the feedstock not only enhances HCl release in gaseous form but also promotes the conversion of heavy metals into more volatile chloride species via chlorine migration and competitive reactions [29]. Furthermore, the conversion rate of ZnO(s) increases slightly with an increased degree of MSW classification. This implies that, despite the overall trend of chlorine-promoted volatilization, a portion of Zn tends to remain in the bottom ash as an oxide. Therefore, refuse classification alters furnace feedstock composition, particularly by increasing chlorine content. This, in turn, enhances HCl volatilization and promotes the chlorination–volatilization of heavy metals. Consequently, controlling chlorine input should be prioritized in practical waste-to-energy or incineration system designs.

### 3.4. Influence of Blending Ratio on Migration Characteristics of Heavy Metals and HCl

**Figure 4:**
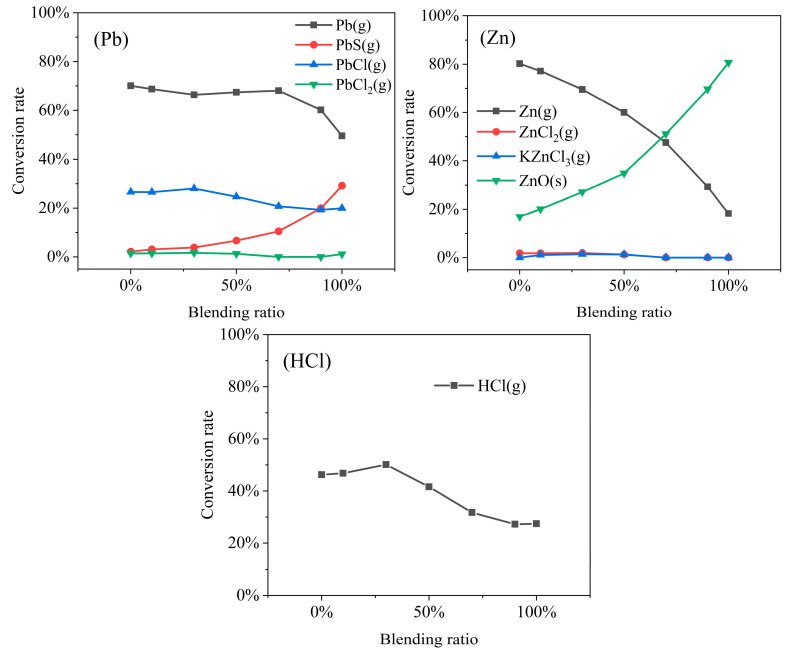
Effect of blending ratio on the equilibrium speciation fractions of HCl and the products of Zn and Pb.

This section examines the influence of the AR blending ratio on heavy metal speciation based on simulation results obtained under operating condition (4). As shown in Figure 4, the AR blending ratio significantly influences the speciation of Zn and Pb as well as HCl volatilization, whereas its effect on Cd, Cr, Cu, and Ni is less pronounced. Figure S4 in the Supplementary Materials provides further details.

For Zn, with increasing blending ratio, the conversion to Zn(g) decreased sharply from 80.2% to 18.2%, whereas the conversion to ZnO(s) increased significantly from 16.9% to 80.7%. This trend may be attributed to the lower moisture content and higher carbon and hydrogen content in AR. Li [9] reported that, during the co-combustion of AR and MSW, heavy metal concentrations in solid residues increased significantly with increasing blending ratios. Meanwhile, conversion to ZnCl_2_(g) and KZnCl_3_(g) remained low and occurred only at low-to-medium blending ratios. When the blending ratio exceeded 70%, these species no longer formed. This is likely because the MSW used in this study contained significantly more chlorine than the AR. Increasing the AR blending ratio reduces the overall chlorine availability in the system, thus limiting the precursors such as HCl(g) or chlorine radicals necessary for volatile ZnCl_2_(g) formation. Wang [34] found, through experiments involving regulation of the chlorine-to-heavy-metal ratio, that a high chlorine content promotes heavy metal chloride formation during MSW combustion. Similarly, the formation of PbCl(g) and PbCl_2_(g) decreased with increasing blending ratio, consistent with the explanation above. At high blending ratios, conversion to PbS(g) increased markedly, whereas conversion to Pb(g) dropped rapidly from 68% to 50% once the blending ratio exceeded 70%. This may be due to the higher sulfur content in AR and the strong affinity between Pb and S. Liu [32] demonstrated PbS formation under high-temperature conditions using tube furnace experiments and thermodynamic equilibrium analysis during sludge combustion. Thermodynamically, PbS(g) is stable as a gaseous species at high temperatures. Botor [35] determined the vapor pressure of PbS across a range of temperatures using multiple techniques. From these measurements, the standard sublimation enthalpy was calculated to be 232.4 ± 0.1 kJ/mol. This indicates that PbS(g) has a substantial vapor pressure at 800 °C. For HCl volatilization, the conversion increased gradually at low blending ratios, peaked at 50.1% at 30% AR, and then decreased steadily at higher ratios. This phenomenon indicates that the effect of AR blending on HCl release does not follow a simple linear relationship. Instead, it involves a process in which modification of the system’s chemical equilibrium triggers complex competitive reactions. These reactions lead to a peak in HCl generation at a specific blending ratio, followed by significant suppression at higher ratios. This study employs a chemical thermodynamic equilibrium model that assumes complete equilibrium, without accounting for reaction kinetics or the physical form of the feedstock. However, chlorine speciation differs significantly between MSW and AR in actual combustion. MSW, especially kitchen waste, contains largely inorganic chlorine, whereas AR is rich in organic chlorine. Organic chlorine is released predominantly at low temperatures, while inorganic chlorine volatilizes mainly at higher temperatures [36]. Therefore, the specific mechanism governing HCl volatilization behavior under AR blending conditions requires further experimental investigation.

### 3.5. Removal Efficiency of Conventional and Modified Calcium-Based Additives

This section examines the simulation results obtained under operating condition 5 to investigate the effects of conventional and modified additives on the removal of heavy metals and HCl during the co-combustion of AR and MSW. As shown in Table 4, the addition of CaO significantly suppressed the volatilization of Zn and HCl but had a relatively limited effect on the volatilization of Cr, Ni, Cu, Cd, and Pb. The products formed from CaO at different temperatures are detailed in Figure S5 of the Supplementary Material. Both modified additives exhibited high removal efficiencies for Cu in the temperature range of 800–1100 °C.

As shown in Table 3, HCl volatilization did not change significantly below 700 °C after CaO addition. This may be attributed to the reaction between HCl and CaO, which forms CaCl_2_ [37]. Additionally, the inherent calcium content in the ash from the raw materials may have reduced the effectiveness of the added CaO. When the temperature exceeded 800 °C, HCl volatilization increased significantly. This was due to the thermal decomposition of CaCl_2_ at high temperatures, which released HCl [38]. Despite this increase, the HCl volatilization rate above 800 °C remained approximately 10% lower than that in the reference case without CaO addition. This reduction can be explained by the fact that, although the reverse decomposition of CaCl_2_ occurs, it does not proceed to completion, so the system reaches a new equilibrium with a lower HCl concentration than in the absence of CaO [39]. Thus, although high temperatures promoted some HCl release, the volatilization rate remained lower than in the absence of CaO. Regarding Zn, its volatilization was partially suppressed by CaO addition in the 700–800 °C range. The mechanism likely involved the reaction of CaO with acidic gas-phase components (e.g., HCl, SO_2_, CO_2_), which altered the composition and properties of the gaseous environment. Based on thermodynamic equilibrium principles, these alterations in the gaseous environment influenced the equilibrium distribution of Zn species, thereby suppressing Zn volatilization [40,41].

As shown in Table 3, in the temperature range of 800–1100 °C, the modified additives CaB_5_SiO_9_(OH)_5_ and CaBSiO_4_OH exhibited high removal efficiencies for Cu. Among them, CaB_5_SiO_9_(OH)_5_ performed best, achieving copper volatilization rates ranging from 2.2% to 25.8% across the tested temperatures. Although the modified additives led to a slight increase in HCl volatilization compared with CaO, their overall HCl removal performance remained effective. In contrast, both modified additives enhanced Zn volatilization to some extent, which was particularly pronounced at 800 °C. This phenomenon may stem from the ability of these additives to subtly alter the local oxygen partial pressure or chlorine distribution within the melt, thereby facilitating the formation of volatile zinc chlorides or elemental zinc [42]. Overall, both modified additives demonstrated a superior capability for immobilizing heavy metals compared to conventional CaO. The key structural modification in these additives is the introduction of boron into a calcium silicate system. Boron typically exists as three-coordinated trigonal BO_3_ units, which act as network modifiers to significantly reduce the polymerization and connectivity of the silicate network. The resulting loose network structure lowers the eutectic point temperature of the system, facilitating the formation of boron-rich silicate hypereutectic liquid phases—either locally or globally—within the 800–1100 °C range [43]. This process has been extensively validated in studies on vitrification stabilization of incinerator fly ash, where B_2_O_3_ addition is recognized as an effective approach to reduce melt viscosity and promote glass formation [44]. Furthermore, this loose network structure provides additional tunable coordination environments and vacancies, facilitating the incorporation of foreign cations with varying ionic radii and charges (e.g., Cu^2+^) [45,46]. Thus, the combined mechanism of these two modifiers reflects synergistic physical and chemical processes. First, the low-eutectic-temperature liquid phase forms at high temperatures, effectively encapsulating and dissolving copper-containing particles and providing physical isolation. Second, more stable fixation is achieved through lattice solid solution and ion-exchange processes. Thermodynamic equilibrium analysis indicates that above 800 °C, CaB_5_SiO_9_(OH)_5_ and CaBSiO_4_OH exhibit significant inhibition effects on the volatilization of Cu. Under these conditions, Cu primarily volatilizes in the form of chloride species. Thus, their stability is primarily influenced by temperature and chemical environment. Both boron-containing additives studied remain stable within the measured temperature range but may decompose into simple oxides at higher temperatures, disrupting their specific structures for inhibiting volatilization [47]. Additionally, an excessively high chlorine content in the atmosphere may trigger reactions between high concentrations of HCl or Cl_2_ and the immobilized copper, resulting in its re-volatilization [48]. Simultaneously, the boron component in the additives is itself susceptible to volatilization under high-temperature, humid conditions [49]. Consequently, future research should focus on two areas: first, thoroughly investigating the volatilization inhibition performance and stability limits of these additives in complex real-world environments, and second, developing composite additives capable of synergistically immobilizing multiple heavy metals at elevated temperatures while efficiently removing HCl.

### 3.6. Ecological Risk Analysis of Heavy Metals in Exhaust Gas

Gaseous heavy metals in flue gas are typically highly toxic. To assess the impact of modified calcium-based additives on the ecological risk posed by heavy metals in exhaust gases, this section evaluates the risks under operating Condition 5 for CaO and the modified additives within the optimal temperature range. Individual potential ecological risk factors and risk indices under different calcium-based additives are presented in Table 5.

The individual potential ecological risk factors and the overall risk index for heavy metals with different calcium-based additives are shown in Table 5. The data show that both the Er values for individual metals and the overall RI were highest in the absence of any additive, indicating that direct release of untreated flue gas could significantly harm the ecosystem. In the absence of additives, Pb, Cd, Cu, and Zn exhibited notably high Er values, whereas Cr and Ni were absent from the gas phase and thus had Er values of zero. In the 800–900 °C range, CaO suppressed Zn volatilization but slightly promoted Cu volatilization; since Cu’s Er value far exceeds that of Zn, the net effect was an increase in the overall RI. In contrast, the modified additives, CaBSiO_4_OH and CaB_5_SiO_9_(OH)_5_, substantially reduced the ecological risk from Cu, thereby lowering the overall RI. In summary, within the 800–1100 °C range, the modified calcium-based additives CaBSiO_4_OH and CaB_5_SiO_9_(OH)_5_ significantly inhibited the ecological risk index of combustion exhaust gases, and their effectiveness was especially notable at higher temperatures.

## 4. Conclusions

This study investigates the release characteristics of heavy metals and HCl during the co-combustion of AR and MSW by employing thermodynamic equilibrium analysis. It also examines the effects of temperature, N_2_/O_2_ ratio, blending ratio, refuse classification degree, and the addition of CaO and modified additives on the volatilization behavior. The conclusions are as follows:

1. During the co-combustion of AR and MSW, Cd and Pb are almost completely volatilized above 400 °C. The volatilization of Cu and Zn increases significantly between 500 °C and 800 °C and is nearly complete above 900 °C. Cr and Ni exhibit low volatility across the entire temperature range, with accumulation primarily occurring in the bottom ash. The volatilization of HCl increases continuously with temperature and accelerates significantly above 700 °C. Excess air promotes partial conversion of Zn, Pb, and Cu products to oxides.
2. At 800 °C, a lower N_2_/O_2_ ratio proves effective in suppressing the volatilization of Zn and the emission of HCl. Higher degrees of MSW classification promote the chlorination of Zn and Pb and the volatilization of HCl but enhance the fixation of Zn. An increased AR blending ratio promotes the fixation of Zn as ZnO(s), whereas Pb is predominantly volatilized as PbS(g).
3. At temperatures above 800 °C, CaO reduces HCl volatilization by approximately 10%. Additionally, at 800 °C, it exhibits a suppressive effect on Zn volatilization. Furthermore, within the temperature range of 800–1100 °C, the modified additives CaB_5_SiO_9_(OH)_5_ and CaBSiO_4_OH demonstrate a strong fixation capacity for Cu and significantly reduce the risk index of the exhaust gases.

## Figures and Tables

**Table 1:** Percentage composition of MSW components based on classification degree.

Classification Degree	Kitchen Waste	Wood and Bamboo	Paper	Rubber and Plastic	Textile
Low	34.27%	13.03%	7.07%	40.35%	5.28%
Medium	23.09%	8.78%	6.17%	53.65%	8.58%
High	10.95%	4.16%	7.87%	64.81%	12.21%

**Table 2:** Input parameters for AR and MSW derived from literature data.

Element	MSW			Aged Refuse [18]
	Low Degree Classification	Medium Degree Classification	High Degree Classification	
C (wt%)	25.17	27.40	29.45	54.11
H (wt%)	3.59	3.99	4.36	8.05
O (wt%)	10.77	9.82	9.25	17.42
N (wt%)	0.62	0.52	0.43	0.8
S (wt%)	0.11	0.11	0.11	0.19
Cl (wt%)	3.27	3.70	4.01	1.79
K (wt%)	0.58	0.46	0.31	0.58
Na (wt%)	0.79	0.62	0.44	0.79
Mg (wt%)	0.45	0.53	0.61	0.45
Ca (wt%)	7.22	8.05	8.73	9.40
Al (wt%)	0.73	0.87	1.06	1.1
Fe (wt%)	0.49	0.45	0.40	0.39
H_2_O (wt%)	46.21	43.47	40.84	4.94
Pb (mg/kg)	42.86	41.72	45.53	39.41
Cd (mg/kg)	2.54	2.70	2.81	2.82
Cr (mg/kg)	189.18	221.84	261.32	283.66
Cu (mg/kg)	83.91	97.72	113.06	123.45
Zn (mg/kg)	299.78	300.59	300.55	327.69
Ni (mg/kg)	80.52	94.78	110.02	121.99

**Table 3:** Comparison of volatilization rates of HCl and heavy metals under different excess air coefficients (λ = 1 and λ = 1.2).

T (°C)	HCl		Pb		Cd		Cr		Cu		Zn	
	λ = 1	λ = 1.2	λ = 1	λ = 1.2	λ = 1	λ = 1.2	λ = 1	λ = 1.2	λ = 1	λ = 1.2	λ = 1	λ = 1.2
300	0.1%	4.0%	0%	99.9%	99.8%	0%	0%	0%	0%	0%	0%	0%
400	1.4%	7.6%	99.9%	99.5%	99.8%	32.0%	0%	0%	0%	46.4	0%	0%
500	7.0%	12.5%	99.9%	99.9%	99.8%	99.8%	0%	0%	0%	99.9%	0%	0%
600	11.6%	18.4%	99.9%	99.9%	99.8%	99.8%	0%	0%	1.4%	99.9%	5.8%	0%
700	18.4%	23.3%	99.9%	99.9%	99.8%	99.3%	0%	0%	35.7%	98.9%	21.1%	21.0%
800	50.1%	40.2%	99.9%	99.7%	99.8%	99.4%	0%	0%	99.4%	99.9%	72.8%	8.2%
900	60.8%	50.7%	99.3%	99.5%	99.8%	98.1%	0%	0%	99.9%	99.9%	93.8%	11.8%
1000	62.1%	51.9%	99.7%	99.9%	99.8%	97.2%	0%	1.1%	99.9%	99.9%	98.9%	10.7%
1100	62.4%	52.1%	99.7%	99.8%	99.8%	97.3%	0%	1.2%	99.9%	99.9%	99.7%	9.3%

**Table 4:** Volatilization rate of heavy metals in the presence of different additives at 800~1100 °C.

Additive	T (°C)	HCl	Cu	Zn
none	800	50.1%	99.4%	72.8%
CaO	800	45.3%	99.4%	64.2%
CaB_5_SiO_9_(OH)_5_	800	45.5%	2.2%	85.9%
CaBSiO_4_OH	800	45.4%	6.3%	81.0%
none	900	60.9%	100.0%	93.8%
CaO	900	51.3%	100.0%	91.7%
CaB_5_SiO_9_(OH)_5_	900	51.6%	6.6%	97.1%
CaBSiO_4_OH	900	51.4%	15.1%	95.7%
none	1000	62.1%	100.0%	98.9%
CaO	1000	52.2%	100.0%	98.4%
CaB_5_SiO_9_(OH)_5_	1000	52.5%	14.1%	99.5%
CaBSiO_4_OH	1000	52.3%	30.0%	99.2%
none	1100	62.4%	100.0%	99.7%
CaO	1100	52.5%	100.0%	99.6%
CaB_5_SiO_9_(OH)_5_	1100	52.7%	25.8%	99.9%
CaBSiO_4_OH	1100	52.7%	34.9%	99.8%

**Table 5:** Individual potential ecological risk factors and risk indices under different calcium-based additives.

Temperature (°C)	Additive	Er						RI
		Pb	Cd	Cr	Cu	Zn	Ni	
800	none	140	2657	0	1477	76	0	4350
800	CaO	140	2657	0	1475	67	0	4340
800	CaBSiO_4_OH	140	2657	0	93	85	0	2975
800	CaB_5_SiO_9_(OH)_5_	140	2657	0	33	90	0	2920
900	none	139	2657	0	1477	99	0	4372
900	CaO	140	2657	0	1487	97	0	4381
900	CaBSiO_4_OH	139	2657	0	225	101	0	3122
900	CaB_5_SiO_9_(OH)_5_	139	2657	0	97	102	0	2996
1000	none	139	2657	0	1487	104	0	4388
1000	CaO	140	2657	0	1487	103	0	4388
1000	CaBSiO_4_OH	140	2657	0	446	104	0	3347
1000	CaB_5_SiO_9_(OH)_5_	140	2657	0	209	104	0	3110
1100	none	140	2657	0	1487	105	0	4389
1100	CaO	140	2657	0	1487	105	0	4389
1100	CaBSiO_4_OH	140	2657	0	518	105	0	3420
1100	CaB_5_SiO_9_(OH)_5_	140	2657	0	382	105	0	3284

## Data Availability

The original contributions presented in this study are included in the article. Further inquiries can be directed to the corresponding author.

## Supplementary Materials

The following supporting information can be downloaded at: https://www.mdpi.com/article/10.3390/molecules30244771/s1, Figure S1. Effect of temperature on equilibrium forms of heavy metals under oxidizing atmosphere at excess air coefficient λ = 1.2; Figure S2. Effect of N_2_/O_2_ ratio on the equilibrium speciation fractions of Cd, Cr, Cu, and Ni products, Figure S3. Effect of refuse classification degree on the equilibrium speciation fractions of Cd, Cr, Cu, and Ni products; Figure S4. Effect of blending ratio on the equilibrium speciation fractions of Cd, Cr, Cu, and Ni products; Figure S5. Effect of temperature (after adding CaO) on the equilibrium speciation fractions of HCl and the products of Cd, Pb, Zn, Cu, Cr, and Ni.
