# Supplementary material for: The Release Characteristic and Removal of Heavy Metal and HCl During Co-Combustion of MSW and Aged Refuse: A Preliminary Study Based on Thermodynamic Equilibrium Analysis
Source: Molecules. 2025 Dec 14;30(24):4771. doi: 10.3390/molecules30244771 (PMC12735922; doi:10.3390/molecules30244771)
Supplement: Supplementary file 1 [file molecules-30-04771-s001.zip › molecules-4005987-supplementary.pdf]

Supplementary Materials for

The Release Characteristic and Removal of Heavy Metal and HCl During Co-Combustion of MSW and Aged Refuse: A Preliminary Study Based on Thermodynamic Equilibrium Analysis

Limei Chen [1,2,*], YaojieWang [1], Yanfen Liao [3] and Xiaoqian Ma [3]

[1] School of Automation, Guangdong Polytechnic Normal University, Guangzhou 510665, China

[2] Building Equipment Information Integration and Control Key Laboratory, Guangzhou 510665, China

[3] School of Electric Power, South China University of Technology, Guangzhou 510640, China

* Correspondence: lmchen@gpnu.edu.cn

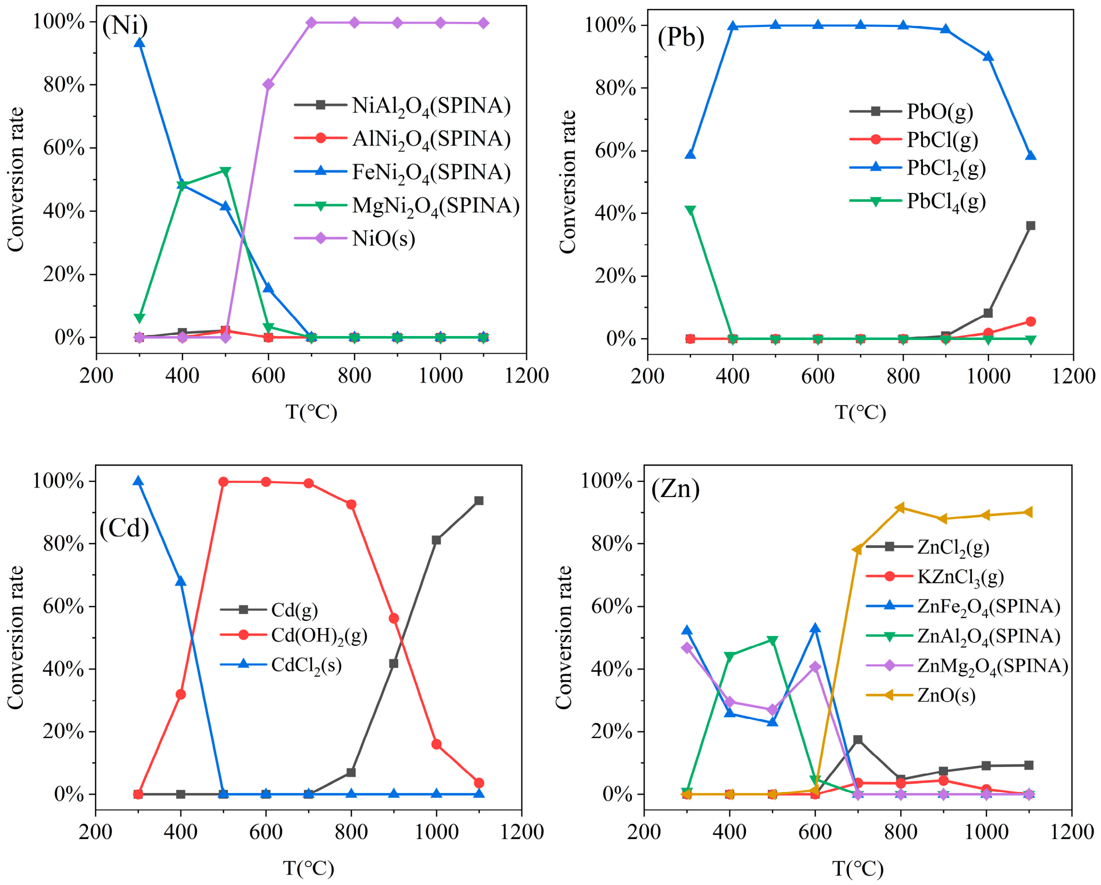

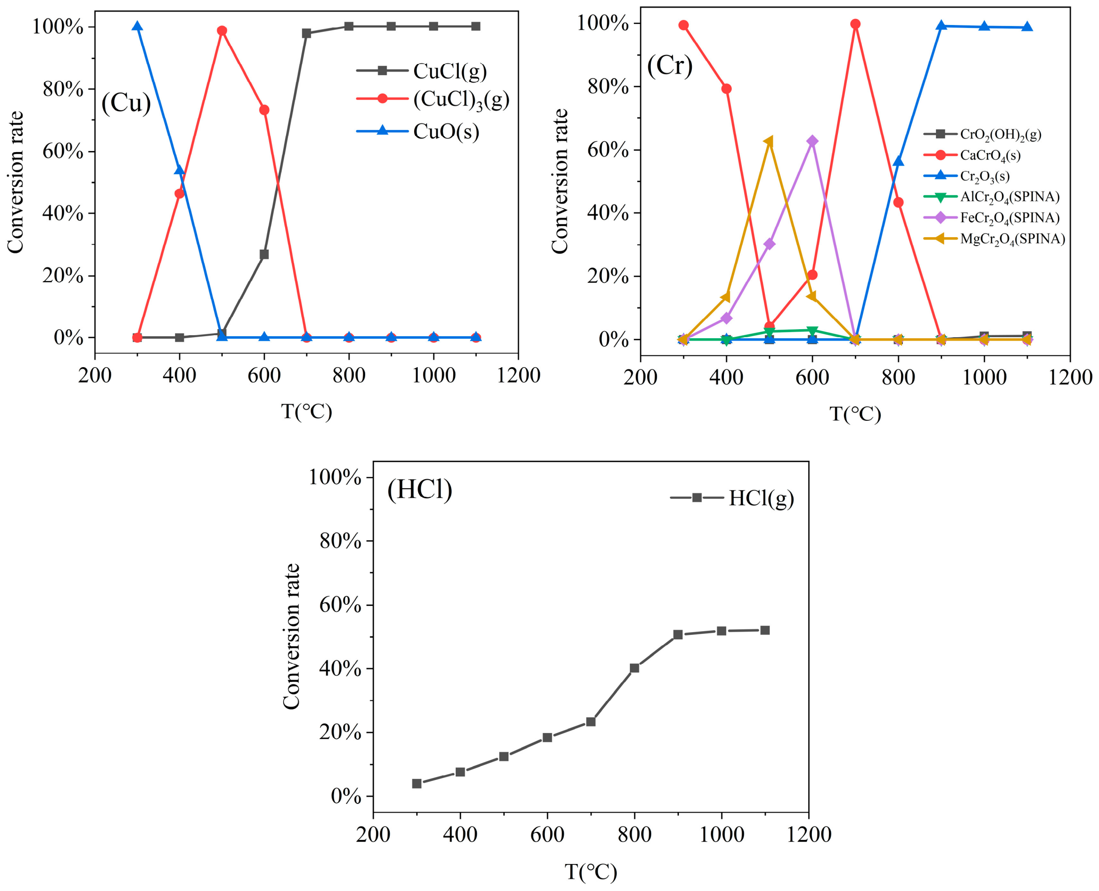

Figure S1. Effect of temperature on equilibrium forms of heavy metals under oxidizing atmosphere at excess air coefficient λ = 1.2.

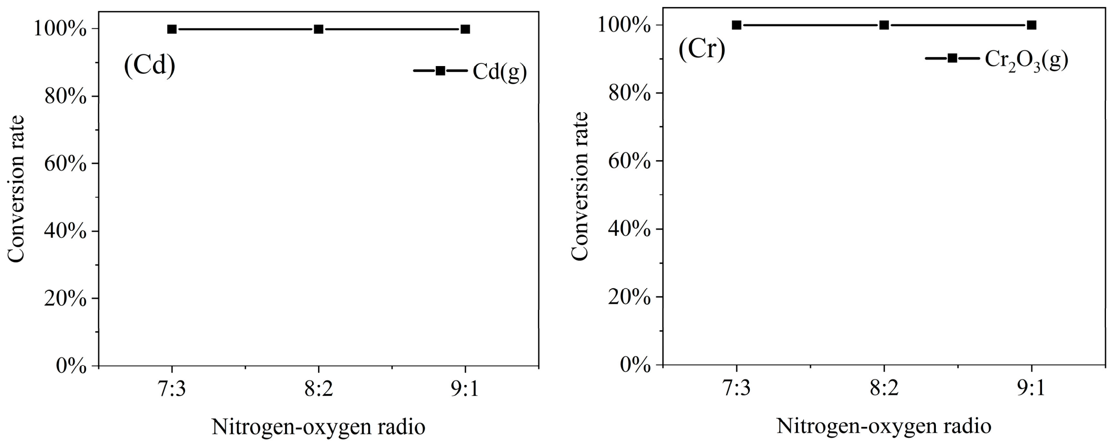

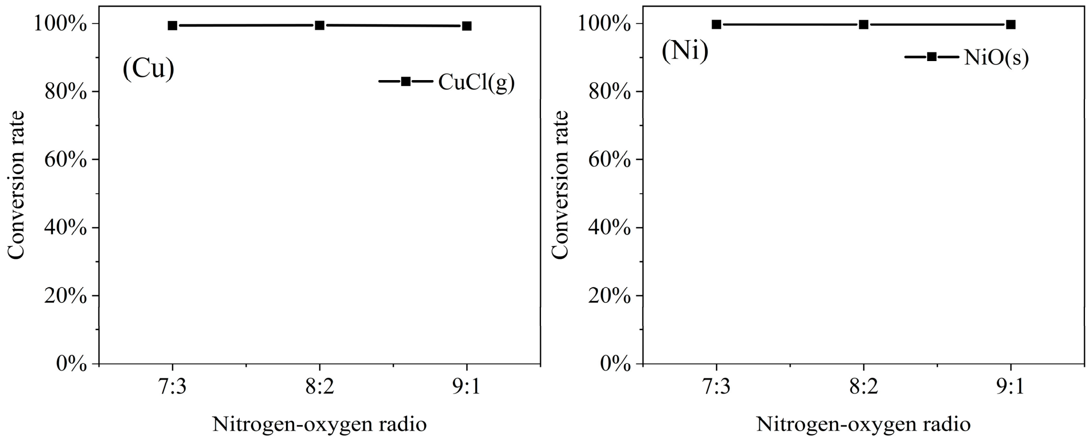

Figure S2. Effect of N_2/O_2 ratio on the equilibrium speciation fractions of Cd, Cr, Cu, and Ni products.

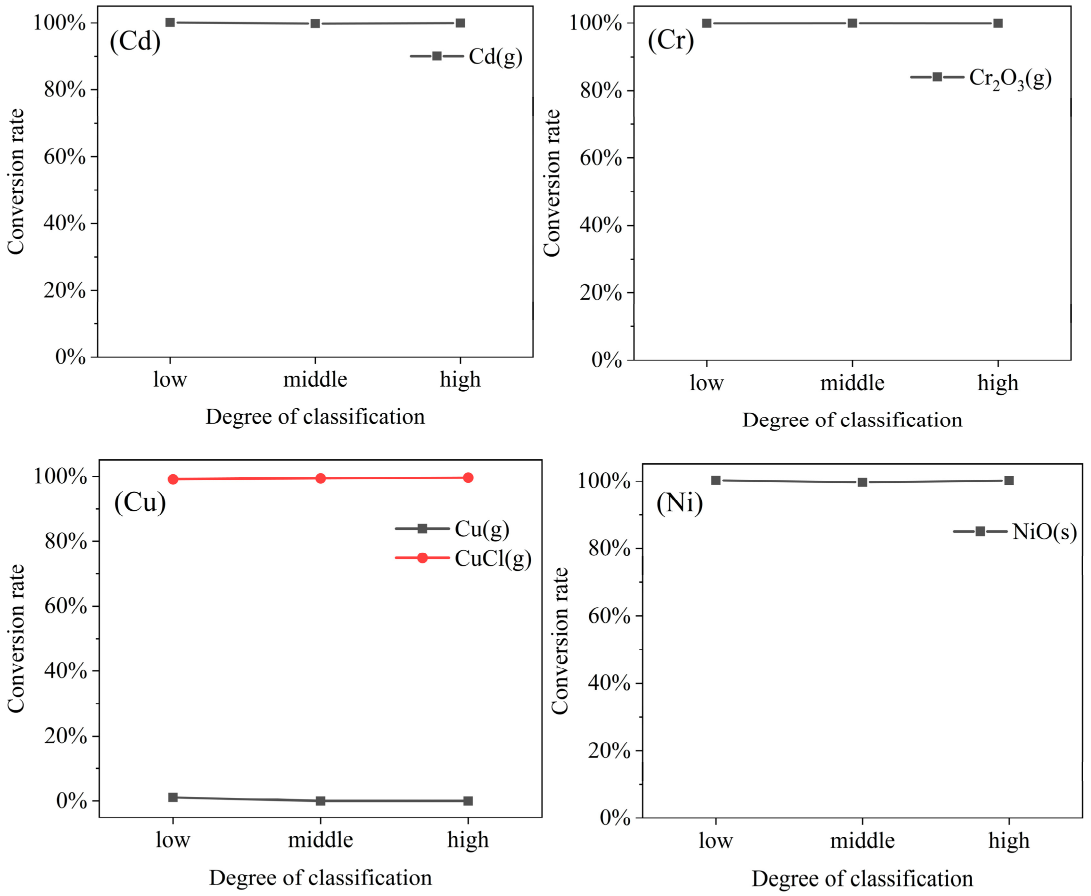

Figure S3. Effect of refuse classification degree on the equilibrium speciation fractions of Cd, Cr, Cu, and Ni products.

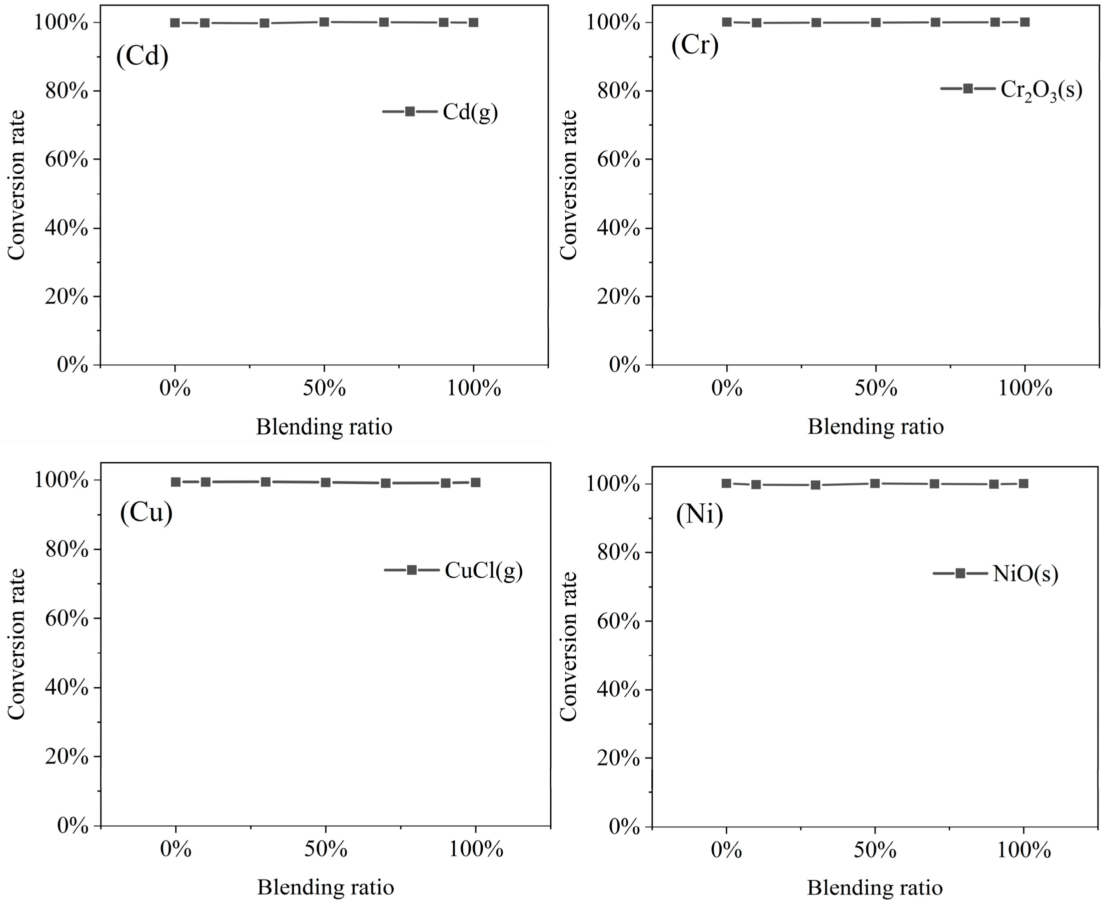

Figure S4. Effect of blending ratio on the equilibrium speciation fractions of Cd, Cr, Cu, and Ni products.

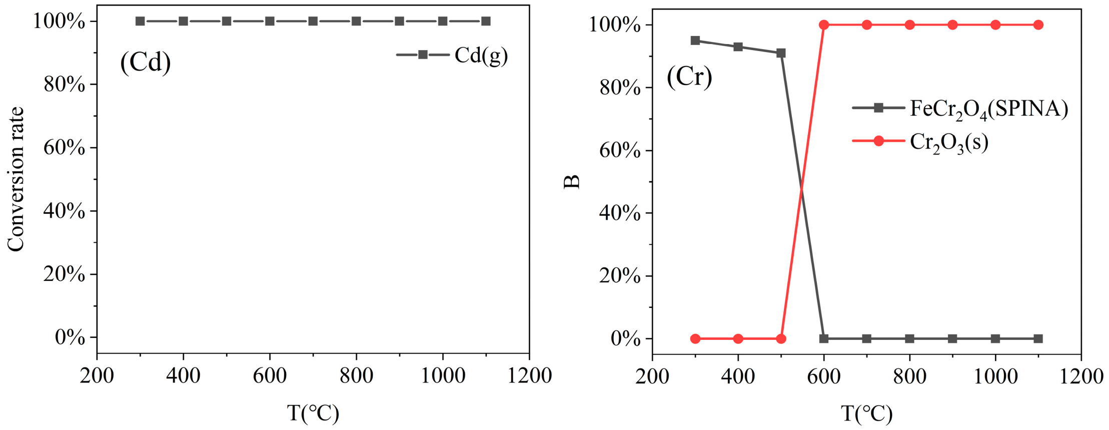

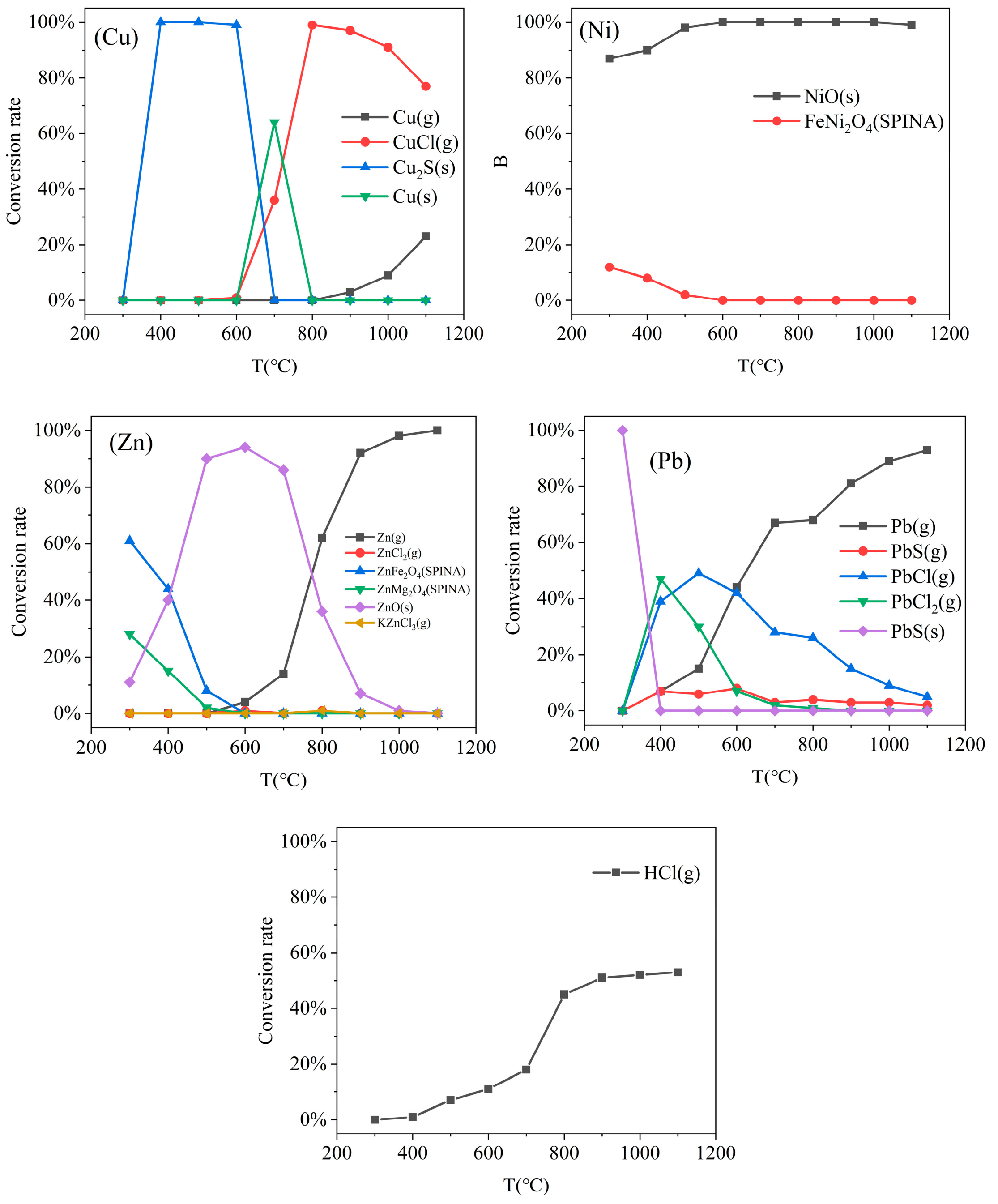

Figure S5. Effect of temperature (after adding CaO) on the equilibrium speciation fractions of HCl and the products of Cd, Pb, Zn, Cu, Cr, and Ni.
